# Electronic nose dataset for COPD detection from smokers and healthy people through exhaled breath analysis

**DOI:** 10.1016/j.dib.2021.106767

**Published:** 2021-01-18

**Authors:** Cristhian Manuel Durán Acevedo, Carlos A. Cuastumal Vasquez, Jeniffer Katerine Carrillo Gómez

**Affiliations:** Multisensor systems and pattern recognition research group, University of Pamplona, North of Santander, Pamplona, Colombia

**Keywords:** Electronic nose, Exhaled breath, COPD, Gas sensor, Machine learning

## Abstract

This article presents a database which was obtained by acquiring measurements through a multisensory device called Electronic Nose (E-nose) based on a matrix of metal oxide sensors, in order to discriminate and classify a group of people affected by the respiratory disease Chronic Obstructive Pulmonary Disease (COPD), smokers and healthy control people through exhaled breath analysis.

The database consists of 4 groups of measurements which were acquired through the E-nose system: 10 control samples (healthy people), 20 samples of people with COPD, 4 samples of smokers and 10 air samples, where in each group two samples of exhaled breath per person were acquired giving a total of 78 samples (40 from COPD, 20 from control, 8 from smokers and 10 from the air)

## Specifications Table

**Table:** 

Subject	Biomedical Engineering; Pulmonary and Respiratory Medicine; Computer Science Applications; Signal Processing
Specific subject area	COPD Detection by using electronic nose technology
Type of data	Text files
Data gathering	Data was acquired using a wireless acquisition card coupled to the E-nose measurement chamber. Exhaled breath measurements were detected by the following sensors: SP-3, MQ-3, TGS 822, MQ-138, MQ-137, TGS 813, TGS 800, and MQ-135. Likewise, a user interface developed in Labview V14 was used to arrange the parameters in the data acquisition.
Data format	Raw data
Parameters for data collection	For the acquisition of the measurement, a protocol was developed for the collection of the breath samples of the people where the samples were taken early in the day and on fasting to avoid errors or possible misunderstanding factors.
	On the other hand, the device was conditioned with a heating system inside the sensor chamber to maintain a constant temperature and relative humidity.
Description of data collection	Data collection was obtained from 3 groups of previously selected people with COPD disease, people with smoking and healthy people.
SOURCE LOCATION	Institution: Amparo san José
	City / Town / Region: Pasto - Nariño
	Country: Colombia
	Latitude and longitude (and GPS coordinates, if possible) for collected samples/data: Latitude: 1°12′47.8"N Longitude: 77°15′50.0"W (1.213281, -77.263883)
	Institution: Guadalupe Foundation
	City / Town / Region: Pasto - Nariño
	Country: Colombia
	Latitude and longitude (and GPS coordinates, if possible) for collected samples/data: Latitude: 1°12′13.7"N Longitude: 77°16′26.8"W (1.203795, -77.274107)
Data accessibility	Repository name: Mendeley Data
	Data identification number: https://doi.org/10.17632/h5pcn99zw4.5
	Direct URL to data: https://data.mendeley.com/datasets/h5pcn99zw4/5
Related research article	C. Cuastumal Vasquez, C. Duran Acevedo, Wireless electronic smell system for the detection of diseases through the exhaled breath, chemical engineering transactions, vol. 68, 2018. https://doi.org/10.3303/cet1868066

## Value of the Data

• The database is a point of reference for the implementation of E-Nose in medical applications [1], [2].
• This database can be useful in the evaluation of different algorithms for classification and pattern recognition and also select which variables are useful for the implementation of the system [3].
• The data will be an important material for the scientific community and society in general to evaluate the capacity of an electronic nose for the detection of COPD, the influence of tobacco on human health as well as for the detection of other respiratory diseases through breath analysis [4].
• The impact of the data and results obtained with the E-nose could encourage society to investigate, develop and implement more strongly this type of device in the health sector since they are an alternative for obtaining rapid and non-invasive for people.

## 1 Data Description

The data were acquired with a sampling rate of 500 samples / second and a resolution of the ADC of 24 bits, obtaining measurements of exhaled breath with a length of 4000 data per sensor through the matrix of 8 sensors or variables (SP-3, MQ-3, TGS 822, MQ-138, MQ-137, TGS 813, TGS-800, MQ-135). In each of the measurements of the three groups analyzed, two measurements were taken per person with the purpose to obtain a correct measurement gathering and also correlate the exhaled breath measurements of the same person (repeatability).

The experiment and evaluation of the e-nose system developed were proposed as a pilot test, therefore, the number of samples was not established as a target since the participation was limited to geriatric centers where volunteers were located in different places, making the samples collection much more efficient. Despite the limitation of the sample size, there was huge cooperation in the geriatric centers for finding the number of COPD cases. It should be clarified that only people over 18 years were involved in this study.

On the other hand, selection and exclusion criteria were made to prevent the samples from being affected by other situations that disturb the volunteers.

### 1.1 Inclusion criteria

• Volunteers over 18 years.
• Patients with a diagnosis of COPD.
• An informed consent form must be signed.

### 1.2 Exclusion criteria

• Patients with related diseases.
• Patients who have been given inhaled medications (nasal route) previously at the time of measurement.

**Fig. 1:**
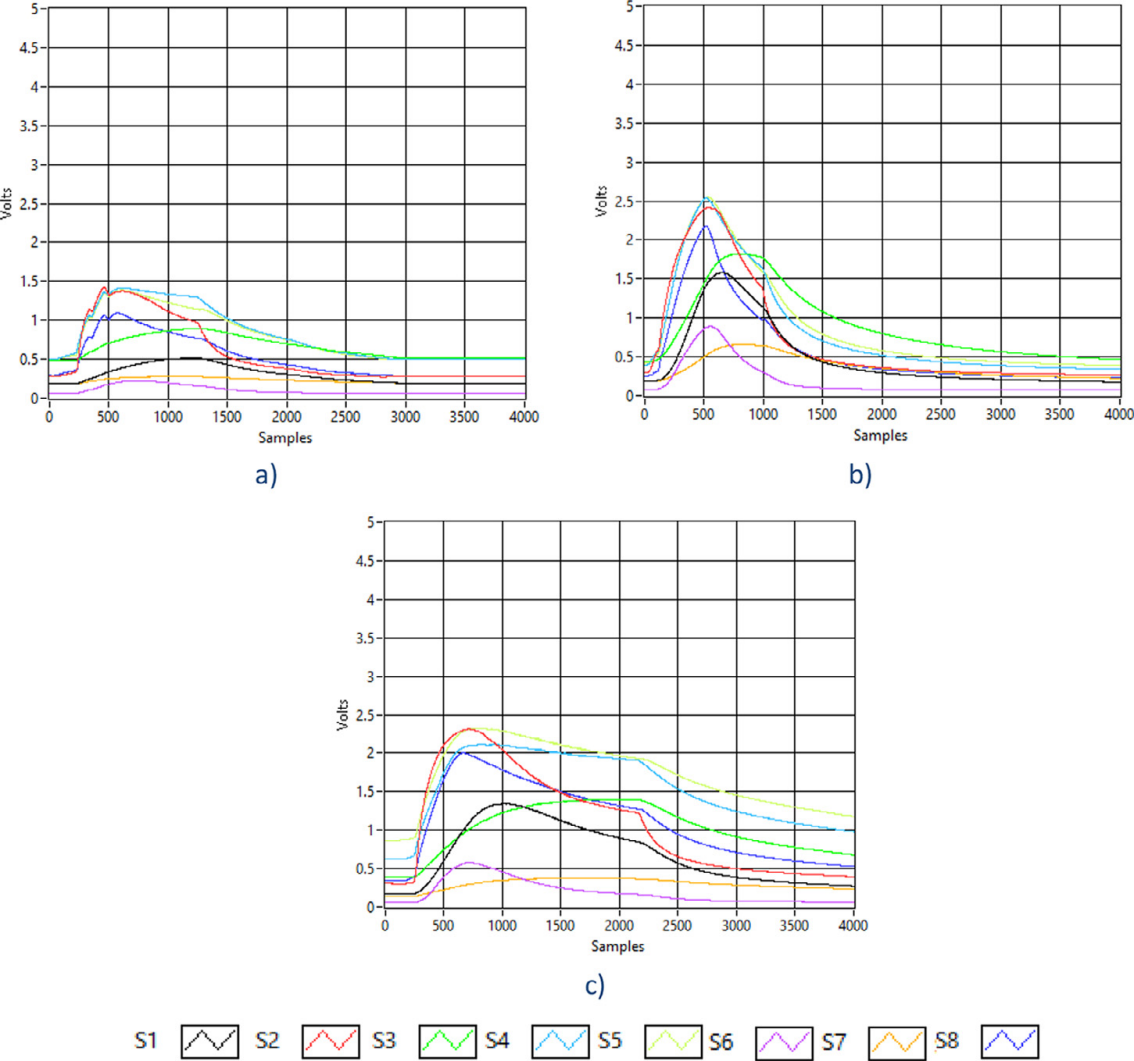
Exhaled breath samples acquired with the e-nose device, S1: SP-3, S2: MQ 3, S3: TGS 822, S4: MQ 138, S5: MQ 137, S6: TGS 813, S7: TGS 800, S8: MQ 135; a) COPD exhaled breath measure, b) Smoker breath measure, c) Control (healthy) breath measure.

In this way, a total of 78 samples were obtained (40 from COPD, 20 from control, 8 from smokers, and 10 from the air. Fig. 1 shows a set of measurements acquired with the E-nose where Fig. 1(a) illustrates a measurement of exhaled breath with the diagnosis of COPD that corresponds to COPD dataset file, Fig. 1(b) depicted a measurement of exhaled breath from a smoker that match to SMOKERS dataset file, and Fig. 1(c) illustrates a measurement of exhaled breath from a healthy person (control) taken as CONTROL dataset file.

The database consists of four files: “COPD.csv” contains the measurements of the 20 patients diagnosed with COPD, “SMOKERS.csv” comprises the measurements of the 4 smokers, “CONTROL.csv” has 10 measurements of healthy persons, and' “AIR.csv” contains the ambient air samples taken during the sampling of each group; the magnitude of the measurements are in units of voltage and the characteristic response of MOS-type sensors in the presence of gas is shown. Additionally, a “General_data_from _the_dataset” file is attached which comprises relevant data such as gender, age, the severity level of COPD (estimated), etc., additionally, for each measurement an identifier was assigned as shown in Table 1.

**Table 1:** Identifier assigned for each exhaled breath measurement from the database corresponding to the COPD, CONTROL, and SMOKERS files.

S#	D or C or S			01	01
Sensors 1 to 8	COPD	CONTROL	SMOKERS	# assigned to each person	# of measurement (2 for each person)

## 2 Experimental Design, Materials and Methods

### 2.1 Experimental setup

**Fig. 2:**
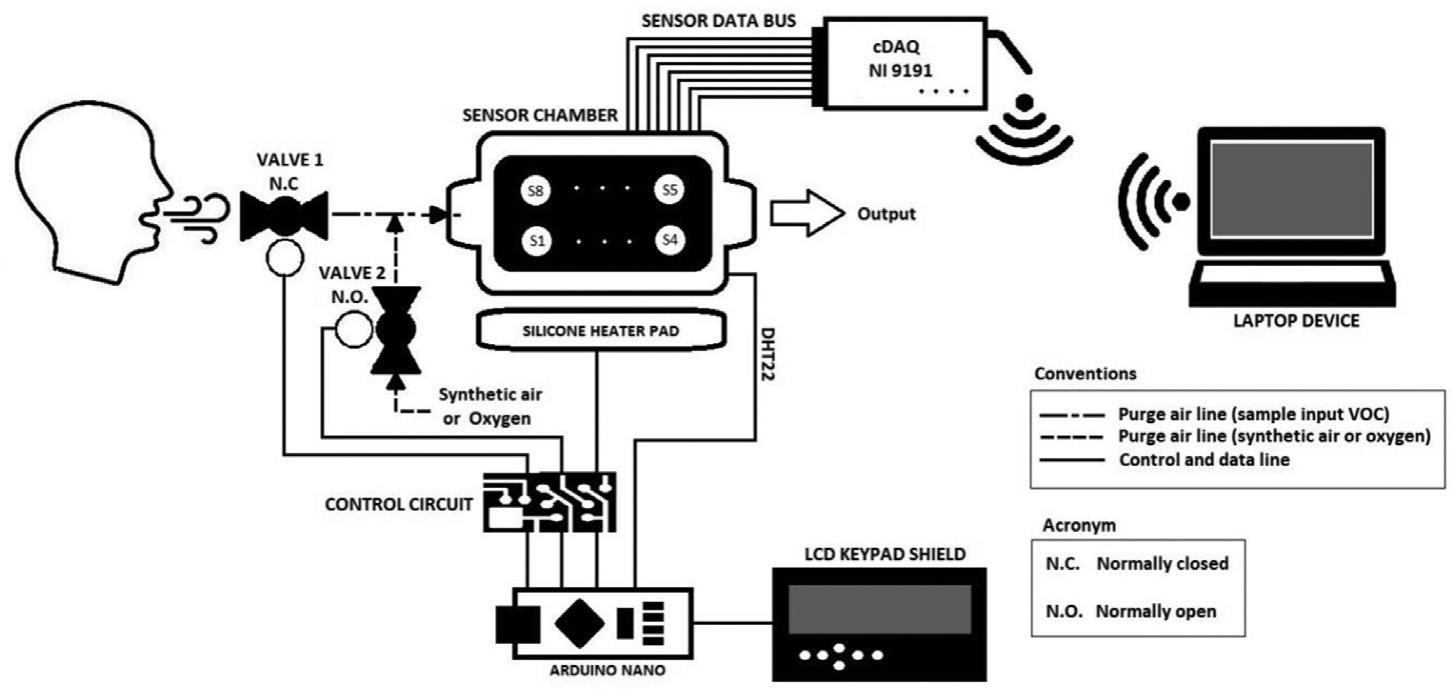
Schematic diagram of E-nose displaying the operation stages.

The E-nose system is made up of a MOS [5], [6] sensor chamber with a capacity of 30 mL, an Arduino nano card to control the electronics, a wireless DAQ card for data acquisition, and a PC where data acquisition software is used and the information is processed and analyzed (See Fig. 2).

For the control of temperature and humidity, a mechanism was implemented in the E-nose sensor chamber to minimize the effect on the responses of the sensors produced by the random change between measurements (exhaled breath) of different people. For this control, a silicone heater pad was used that heats the sensor chamber between 65 °C–70 °C, where it is possible to minimize humidity and condensation inside the chamber keeping it between 0% RH–10% RH [7]. Additionally, the device contains a chamber recovery and cleaning system injecting synthetic air or oxygen by controlling two pneumatic valves (Valve 1 and Valve 2), where valve 1 controls the inlet of samples to the chamber, and valve 2 controls the intake of synthetic air or oxygen in the recovery stage. During this recovery stage, the sensors return to their initial state (based line), and traces of residual VOCs are expelled into the sensor chamber.

### 2.2 Measurement protocol

For the acquisition of breath samples with the e-nose system, a protocol was defined for the participants, where the participant must be fasting before the measurement acquisition to minimize the error crossing due to odors or ingested substances that could be combined with the exhaled VOCs. For this reason, the data acquisition was carried out during the first hours of the day (i.e., 7 am–8 am). Besides, the acquisition time was about 3 min.

Once the e-nose system triggers the sensors and the sensor chamber reaches the set temperature, the patient must place the mouth on the disposable mouthpiece installed at the inlet of the device, since the flow path of the device does not generate a greater make effort to exhale. In this study it was not necessary to use a nose clip, for this reason, it was reported to avoid taking a deeper inhalation before the moment of exhaling to maintain a higher concentration of VOC during exhalation.

The acquisition is made once the operator presses the button acquisition of the device that in turn emits a beep that indicates to the patient the moment to start exhaling. Through the exhalation process, the sensor array performs the reading of the entered VOCs in the sensor chamber by the volunteer whereas data are recorded by assigning an identifier as established (Table 1) and saving it into a CSV file. For exhalation, the participant is asked to perform it continuously and with a flow that is comfortable for the volunteers, and the duration depends on the person as the vital capacity varies according to age, therefore, the exhalation can be done when the participant reaches to empty his lungs making a slight effort by contracting the belly to expel the highest amount of VOC housed in the lungs, obtaining the end-tidal.

The acquisition and exhalation process finishes when the participant empties their lungs by making a slight effort by contracting the belly to expel the highest amount of VOC stored in the lungs.

Once the acquisition is finished, the device enters a purging or cleaning process entering synthetic air or oxygen under pressure to clean the chamber and return the sensors to their initial state (based line).

## Ethics Statement

Based on the basic principles of the Declaration of Helsinki and with the prior approval of the ethics committee of the University of Pamplona, the exhaled breath samples were acquired with prior approval of the participants and authorization of the doctor in charge. Each participant was given an informed consent document explaining the procedure to be carried out (non-invasive) and where there would not be any subsequent side effects. Besides, personal information and medical history were protected for the use of the study.

## Declaration of Competing Interest

The authors declare that they have no known competing financial interests or personal relationships which they have, or could be perceived to have, influenced the work reported in this article.

## Data Availability

Dataset_COPD_SMOKERS_CONTROL_AIR (Original data) (Mendeley Data).
